# Roll-to-Roll (R2R) High-Throughput Manufacturing of Foil-Based Microfluidic Chips for Neurite Outgrowth Studies

**DOI:** 10.3390/mi16060713

**Published:** 2025-06-16

**Authors:** Nihan Atak, Martin Smolka, Anja Haase, Alexandra Lorenz, Silvia Schobesberger, Stephan Ruttloff, Christian Wolf, Ana Ayerdi-Izquierdo, Peter Ertl, Nerea Briz Iceta, Jan Hesse, Martin Frauenlob

**Affiliations:** 1 Joanneum Research Forschungsgesellschaft mbH-MATERIALS, Franz-Pichler-Strasse 31, 8160 Weiz, Austria; nihan.atak@joanneum.at (N.A.); martin.smolka@joanneum.at (M.S.); anja.haase@joanneum.at (A.H.); stephan.ruttloff@joanneum.at (S.R.);; 2 Faculty of Technical Chemistry, TU Wien, Getreidemarkt 9, 1060 Vienna, Austria; alexandra.lorenz@tuwien.ac.at (A.L.); silvia.schobesberger@tuwien.ac.at (S.S.);; 3 TECNALIA, Basque Research and Technology Alliance (BRTA), Mikeletegi Pasealekua 2, 20009 Donostia-San Sebastián, Spain; ana.ayerdi@tecnalia.com (A.A.-I.);

**Keywords:** brain-on-a-chip, foil-based microfluidics, neurite outgrowth, roll-to-roll manufacturing

## Abstract

Microfluidic devices have emerged as a pivotal in vitro technology for axon outgrowth studies, facilitating the separation of the cell body from the neurites by geometric constraints. However, traditional microfabrication techniques fall short in terms of scalability for large-scale production, hindering widespread application. This study presents the development of foil-based cell culture chips, made of polyethylene terephthalate and in-house formulated ultraviolet curable liquid resin by high-throughput roll-to-roll (R2R) manufacturing. Here, two microchannel designs were tested to optimize manufacturing quality and assess the neurite outgrowth behavior. The fabricated neuron-foil chips demonstrated biocompatibility and supported neurite outgrowth within microchannels under static cell culture conditions. Furthermore, fluidic flow, oriented either perpendicular or parallel to the microchannel direction, was applied to enhance the biological reproducibility within the neuron-foil chips. These findings suggest that R2R manufacturing offers a promising approach for the high-throughput production of biocompatible microfluidic devices, advancing their potential application in modeling neurological diseases within the biomedical industry.

## 1. Introduction

Nowadays, one in every three individuals is suffering from neurological conditions, which is still the major reason for physical disabilities throughout the world [1,2]. These conditions arise from various factors, such as brain damage (stroke), diseases (Alzheimer’s Disease), or injuries (accidents, traumas) [3], ultimately resulting in neuronal dysfunction. When the neuronal tissues are damaged, the recovery rate is incredibly low and often non-existent, including the inability to reestablish axonal connections properly [4]. Although the peripheral nervous system possesses some regenerative capacity, the central nervous system lacks such ability [4,5], making it even more important to study axonal regeneration and neuron cell communication within the central nervous system [3,6].

Conventional in vitro methods have limitations when it comes to accurately mimicking the in vivo microenvironment for axonal communication on a cellular level [3,7]. Therefore, microfluidics has emerged as a powerful in vitro technology due to smaller working volumes and a more controllable microenvironment. By mimicking the physiological parameters (such as shear stress or fluid flow), it allows us to study axonal guidance and regeneration more closely [8,9,10,11,12]. Numerous studies have focused on axonal isolation from neuronal cell bodies for different applications like axotomy [13], drug screening, and cell differentiation [14,15,16]. Thereby different microfluidic device designs and different cell sources like dorsal root ganglion cells [12,17], cortical neurons [18], stem cells [3,15], neuroblastoma [6,14], and glioblastoma cell lines [19,20] were used. On the other hand, the choice of material is still limited compared to the number of different available cell types. The very first microfluidic device that investigated neurite outgrowth was made of Polytetrafluoroethylene [21]. Polydimethylsiloxane (PDMS) has long been favored in microfluidic device fabrication due to its advantages, including gas permeability, biocompatibility, chemical inertness, and transparency [22,23,24,25,26]. However, PDMS exhibits several limitations, such as low mechanical strength and high hydrophobicity, that hinder its broader application, especially for high-throughput applications, namely the mixing of two liquid compounds, leading to a high risk of bubble formation [27,28,29]. In light of this challenge, thermoplastic polymer materials, however, like polystyrene (PS) [30], polyethylene terephthalate (PET) [5,31], and cyclic olefin polymers [32,33] are gaining attention as alternative materials.

While soft lithography is widely used, other fabrication methods based on thermoplastic polymers [31,34] are gaining popularity since they offer more flexibility in upscaling and high-throughput manufacturing, enabling faster and more straight-forward translation from academia to industry [33,35,36,37]. Among other industrially relevant manufacturing techniques, the Roll-to-roll (R2R) method has gained interest in biological applications, including biosensors and diagnostics assays [31,32,35,38,39,40]. This continuous process offers scalability and cost-effectiveness necessary for production in the medical device industry but is limited to the use of flexible materials and challenges such as air entrapment or material alignment [41,42]. Although already used in diagnostics [31], microfluidic chips that are imprinted and also laminated with the R2R method are not yet used in commercially available cell culture ware.

**Figure 1:**
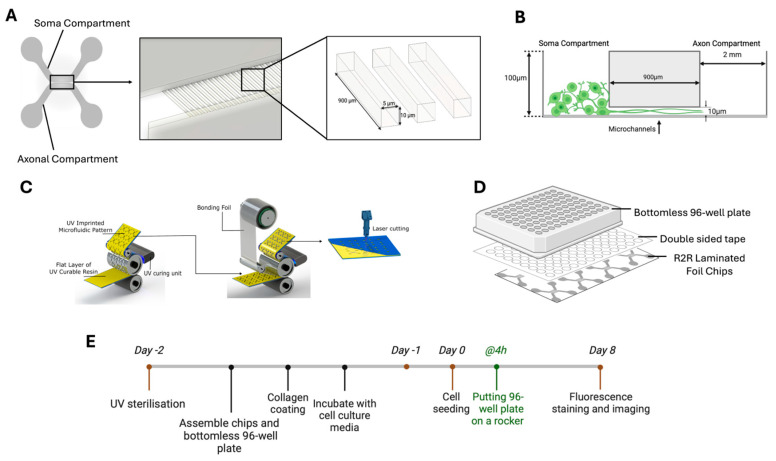
Schematic of the neuron-foil chips design, fabrication, and cell culture application steps. (**A**) Schematic of a single microfluidic chip and magnification of the microchannels area and geometry. (**B**) Cross-sectional view of the main compartments for soma and axon, and the neurite outgrowth inside the microchannels. (**C**) Roll-to-Roll (R2R) UV-NIL imprinting, lamination, and neuron-foil chips’ inlet cutting with CO_2_ laser, respectively. (**D**) Sketch for assembled layer stack of bottomless 96-well plate, double-sided tape, and R2R laminated chips. (**E**) Detailed timeline of handling the neuron-foil chips for neuron cell culture.

This study aims to develop a high-throughput R2R production strategy to fabricate foil-based microfluidic devices with different channel geometries that allow reproducible neurite outgrowth cell studies (Figures S1 and S2) [7,13,43]. The introduced neuron-foil chip (consists of soma (cell body) and axon (neurite outgrowth) compartments (100 µm depth) connected by 70 microchannels (900 µm length, 5 µm width, 10 µm height, Figure 1A) in parallel which should lead to neurite outgrow and keeps the cell bodies separated through size-based geometric constraints (Figure 1B). Two microchannel design variations, *angled* and *straight*, were tested, featuring junction connections of 70° and 90°, respectively, between the microchannel and the soma/axon compartments. The 70-degree angle was incorporated to investigate the impact of channel design on the removal of trapped air during the UV imprinting step. For Manufacturing, this study utilizes a custom-built roll-to-roll (R2R) machine for ultraviolet nanoimprinting lithography (UV-NIL) for large-scale fabrication of foil-based microfluidic devices and a custom-made UV-curable resin, *NILCure 31* [35,44,45]. First, in the imprinting step, the liquid resin was coated onto a transparent PET foil substrate structured by rolling on the nickel shim while curing the resin via UV irradiation (Figure 1C). Second, in the lamination step, the fluidic patterns were enclosed again using the R2R machine by laminating the imprinted layer to a second PET foil coated with the same UV-curable resin. Subsequently, the inlets and array sizes were cut using a CO_2_ laser cutter to fit eight chip units into the well-plate design utilized in conventional cell culture. The fabrication process was monitored by testing the reproducibility and accuracy of the microchannel geometries, air entrapment, and the lamination of the PET foils without collapsing the channels. For cell culture feasibility tests, the neuron-foil chips were bonded to bottomless 96-well plates with double-sided adhesive tape (Figure 1D). U87-MG cells, a glioblastoma cell line, were seeded into the UV-sterilized and collagen type-1-coated soma compartments to direct the neurite outgrowth into the microchannels (Figure 1E). Then, the performance of the neuron-foil chip with *straight* and *angled* channel connections was evaluated by comparing the neurite and cellular morphologies under one static and two dynamic cell culture conditions to the previous studies in the literature, ultimately demonstrating the relevance of R2R manufacturing for neurite outgrowth applications [7,13,43].

## 2. Materials and Methods

A full list of the reagents and devices that were used in this paper can be found in the Supplementary Document, Table S2.

### 2.1. Microfluidic Chip Fabrication: Roll-to-Roll (R2R) Imprinting and Lamination

#### 2.1.1. Microfluidic Design and Simulation

The microfluidic design to produce the nickel shim for R2R manufacturing was created via Autodesk AUTOCAD software (version U.100.M.315, AutoCAD 2024.1), and functionality was simulated via COMSOL Multiphysics software (version 6.3, See Figures S1 and S2).

#### 2.1.2. Roll-to-Roll (R2R) Imprinting

**Figure 2:**
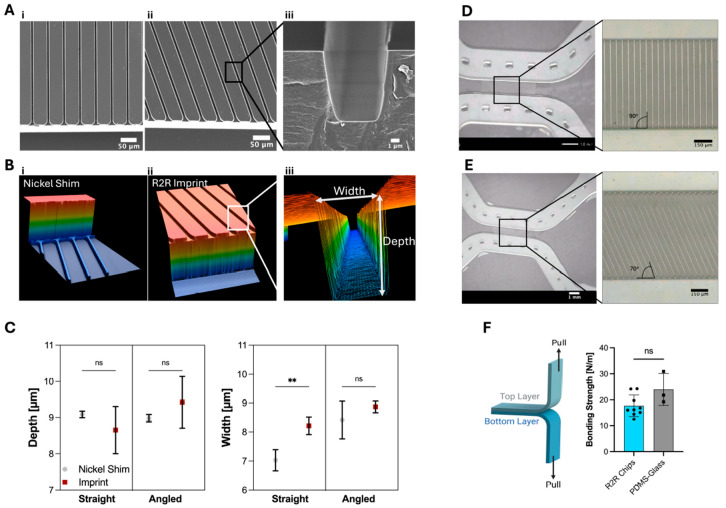
Characterization of the manufactured neuron-foil chip. (**A**) SEM images of the top view of (**i**) *straight* and (**ii**) *angled* microchannels (Scale bars: 50 µm) and (**iii**) the cross-sectional area of a single *angled* microchannel (Scale bar: 1 µm). (**B**) Laser scanning microscopy 3D profiles of *angled* microchannels on (**i**) nickel shim, (**ii**) R2R UV-NIL imprint, and (**iii**) the cross-sectional area of a single microchannel, where white arrows define ‘width’ and ‘depth’ (sample size is 205 µm × 275 µm). (**C**) Comparison of width and depth measurements of microchannels on nickel shim and R2R imprint. Each data point shows the mean ± SD (*n* = 4). (**D**,**E**) Phase contrast microscope image of the laminated areas between *straight* (90^°^) and *angled* (70^°^) microchannels (scale bars: 1mm for the left column and 150 µm for the right column). (**F**) Sketch of bonding strength peel test principle (left) and graph for bonding strength comparison between neuron-foil chips and PDMS-glass chips (*n* = 3–9). Each data point represents a single chip measurement. Ns: non-significant, ** *p* < 0.001 (**C**,**F**).

The imprinting process was adapted from Götz et al. [44]. Briefly, R2R UV nanoimprinting was conducted with the custom-built R2R machine (obtained from Coatema Coating Machinery GmbH, Dormagen, Germany). A PET foil (Melinex^®^ ST506, Pütz GmbH + Co. Folien KG, Taunusstein, Germany) with a width of 28 cm and a thickness of 125 μm was used as a transparent thermoplastic substrate. The *NILCure 31* (Joanneum Research Forschungsgesellschaft mbH) resin was coated onto the surface of the PET foil by slot-die coating. Then, the coated foil substrate comes into contact with a nickel shim wrapped around a magnetic roll, the resin fills the structures and is simultaneously crosslinked with an LED UV lamp at a wavelength of 365 nm (10.4 W/cm^2^). The nickel shim, as a mold to imprint structures into liquid UV-curable resin, has dimensions of 630 mm × 280 mm × 250 μm (Length × Width × Thickness) and was obtained from temicon GmbH, Dortmund, Germany. The detailed channel geometries of the imprint structure for the neuron-foil chips are found in Document S1 (see Figure S1). The web speed, which is the velocity of the foil substrate, was 0.5 m/min, and all the steps were performed in a continuous mode. The geometries of the resulting R2R imprints were subsequently analyzed via scanning electron microscopy (Figure 2A) and confocal laser microscopy (Figure 2B) and compared to the geometries on the nickel shim (Figure 2C).

#### 2.1.3. Roll-to-Roll (R2R) Lamination

To close the R2R structures in a microfluidic chamber, a lamination process was performed with the same *NILCure 31* resin on a 75 µm-thick PET foil (Melinex^®^ ST506), named lamination foil. First, the foil was coated with a thin layer of UV-curable resin (between 1 and 2 µm) and partially cured with a UV LED light source. Then, the lamination foil was wound onto an empty roll core. Later, both rolls, the roll with the R2R imprinted structures and the roll with lamination foil, were attached to the R2R machine. In the machine, the lamination foil and R2R imprinting foil come in contact by applying a 2-bar counter-pressure at the UV curing unit. Through the counter-pressure and the UV initiation, the surfaces of the resin-coated foils chemically crosslink and thereby seal the microfluidic channels. The pillars in the soma and axon compartments of neuron-foil chips were included in the design to prevent the collapse of channels under 2 bar counter-pressure. All the layers are cured with an LED UV lamp with the power of 13 W/cm^2^ (365 nm) simultaneously. The inlets of the bonded neuron-foil chips were subsequently cut via a laser cutter (Speedy 400, Trotec, Marchtrenk, Austria) and additionally cut so that an array of 4 × 2 chips fit onto the bottom of a standard well-plate format used in cell culture applications. To monitor the lamination process, phase contrast microscopy (Figure 2D,E) and the bonding strength between lamination and R2R imprint foil of the final neuron-foil chips were analyzed (Figure 2F).

#### 2.1.4. Material Characterization via SEM, Confocal Laser Microscopy, and Tensile Testing

For SEM, the fabricated imprints were cut via the laser cutter for the cross-sectional view, sputter-coated with gold, and observed at a magnification of 150× with the JEOL JSM-IT100 (JEOL Ltd., Tokyo, Japan). For 3D laser scanning microscopy (KEYENCE INTERNATIONAL (BELGIUM) NV/SA, VK-X1050, Mechelen, Belgium), the imprints were observed as is at a magnification of 50× for a sample size of 205 × 275 × 150 µm (W × L × D). The bonding strength of the finalized laminated neuron-foil chip was measured via a peel test on an Instron 3342 tensile tester (Norwood, MA, USA). Here, the contact area between substrates was 26 mm × 26 mm, with a sample size of 75 mm × 26 mm, and was pulled apart at a velocity of 0.06 m/min.

### 2.2. Cell Culture and Maintenance

Unless otherwise noted, all cell culture reagents and culture ware are purchased from Sigma-Aldrich and are used as purchased. The glioblastoma cell line U87-MG and the neuronal stem cell line NE-4C were obtained from the Biobank at the Medical University of Graz, Austria, and ATCC^®^, respectively. The U87-MG cells were utilized for the on-chip culture studies on neurite outgrowth, whereas the NE-4C cells were utilized for the cytotoxicity studies because this cell line is known to be more susceptible to cytotoxic effects than glioblastoma cells [46]. Cytotoxicity testing is described in Figure S4. The cells were cultured in DMEM High Glucose media, 10% FBS (heat-inactivated, Corning, NY, USA), 1% Antibiotic/Antimycotic solution, and 1% MEM-NEAA (complete DMEM) unless stated otherwise. The media was changed on the first day after thawing, then every 2 days, and cells were split using Trypsin-EDTA 0.5% when reaching 85% confluency until seeding in the neuron-foil chips. The U87-MG cells were maintained in the incubator (CellXpert^®^, C170i, Eppendorf, Hamburg, Germany) at 5% CO_2_, 95% humidity and 37 °C.

### 2.3. Cell Culture on Neuron-Foil Chips

As displayed in Figure 1E on day −2, the channels of the neuron-foil chip arrays (4 × 2 chips) were filled with EtOH 70% and sterilized in a UV chamber at 60 °C until the EtOH evaporated. Then, the neuron-foil chip arrays were attached to bottomless 96-well-plate microfluidic devices via a double-sided adhesive (Microfluidic ChipShop, Jena, Germany) in a laminar flow hood, washed with phosphate buffer saline (PBS), and to allow cell attachment the channel surfaces were coated with collagen type-1 from rat tail (Sigma-Aldrich, St. Louis, MO, USA) for 24 h. Until cell seeding on day 0, chips were incubated at 37 °C in a humid atmosphere and washed with PBS before the seeding step with U87-MG cells used in the neurite outgrowth studies.

On the day of cell seeding (day 0), a cell solution with a density of 1.25 × 10^6^ cells/mL in complete DMEM was prepared. Notably, 20 µL from that solution was seeded into the soma compartment of the neuron-foil chips and maintained for 2 h in the incubator for cell attachment. When the cells adhered onto the surface, 350 µL and 250 µL of complete DMEM were added to the inlet and outlet wells of the axon and soma compartments, respectively, to allow a flow-driven removal of unattached cells based on hydrostatic pressure.

For the static cell culture condition, complete DMEM was exchanged every 2 days by aspiration of the culture media and the addition of 350 µL and 250 µL in the inlet and outlet wells of axon and soma compartments to initiate a quick hydrostatic pressure-driven exchange of culture media in the channels.

For the dynamic cell culture, complete DMEM was exchanged as described for the static culture, and these neuron-foil chip arrays were placed on a rocker (VWR, 444-0759, Avantor^®^, NJ, USA) inside the incubator. The rocker setting was set to ±2° tilting in continuous mode at a tilting speed of 1 rpm. For the condition of lateral flow, the arrays were oriented on the rocker so that the maximum hydrostatic pressure difference was created between the inlet and outlet compartments, mainly initiating flow within each soma and axon compartment. For the flow-through condition, the well-plate orientation was rotated 90° so that the maximum hydrostatic pressure difference is created between the axon and soma compartments to generate flow through the microchannels for neurite outgrowth. All neuron-foil chip conditions were cultured up to day 8, where neurite outgrowth, cell area measurement, and the relative number of neurites per chip were analyzed.

### 2.4. Fluorescence Microscopy Imaging on Neuron-Foil Chips

On day 8, the cells in the neuron-foil chips were fixed in 4% paraformaldehyde in PBS containing Ca^2+^ and Mg^2+^ (PBS+) for 20 min at room temperature and permeabilized with 0.2% Triton X-100 in PBS+ for 5 min at RT. Later, cells were blocked with 5% BSA in PBS+ for 1h at RT. The counterstain of Phalloidin-iFluor 555 (Abcam, 1:1000, Cambridge, UK) and Hoechst (Sigma-Aldrich, St. Louis, MO, USA, 1:500) was used at room temperature for 1 h. Before every step, washing with PBS+ was performed three times. At least three representative images per chip were acquired with a fluorescence microscope (Olympus, IX83) and processed with ImageJ (version 1.53k) and Fiji (version ImageJ2, 2.16.0/1.54p) software. Images for neurite outgrowth, cell area measurement, and the relative number of neurites per chip were processed and analyzed as follows.

**Figure 3:**
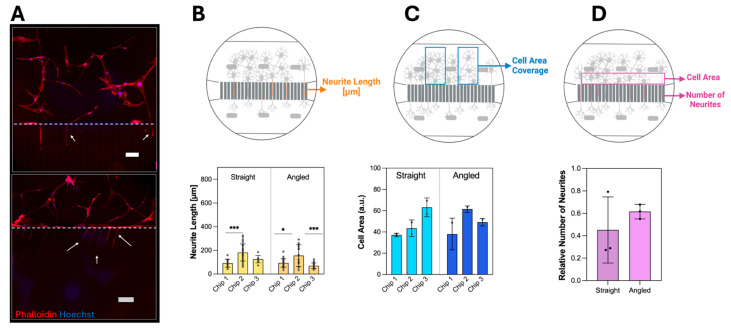
U87-MG neurite outgrowth and cell morphology investigation with different microchannels after 8 days of static cell culture. (**A**) F-actin (Phalloidin, red) and nuclei (Hoechst, blue) fluorescence imaging under static culture conditions for the *straight* and *angled* different microchannel designs (Scale bars: 100 µm). Schematic drawings and data visualization for neurite length (**B**), grey dots represent lengths of single neurite), region of interest for cell area coverage (**C**), and relative number of neurites (**D**) (*n* = 3 biological replicates, bars represent mean values ± SDs, * *p* < 0.05, *** *p* < 0.001).

**Figure 4:**
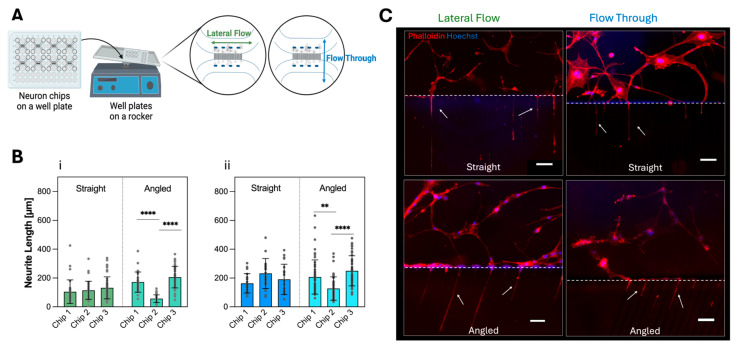
U87-MG neurite outgrowth investigation with two different microchannel designs after 8 days of dynamic cell culture. (**A**) Sketch of 8 neuron-foil chips bonded to a bottomless 96-well plate, placement onto a rocker to initiate lateral flow and flow-through conditions in the channels. (**B**) The neurite length comparison for (**i**) lateral flow and (**ii**) flow-through for *straight* and *angled* microchannel designs (Bars represent the mean values ± SDs, *n* = 3 biological replicates, dark grey dots represent lengths of individual neurites). (**C**) Fluorescence imaging of F-actin (red) and nuclei (blue) inside the *straight* and *angled* microchannel chips in lateral flow and flow-through conditions (Scale bars: 100 µm. ** *p* < 0.01, **** *p* < 0.0001).

Neurite Outgrowth Quantification: Axon lengths were measured from the fluorescence images (Figure 3 and Figure 4). Firstly, the scale bar was set on the Fiji software, and the images were converted to 8-bit and saved in TIFF format. Afterward, the NeuronJ plugin (version 1.4.3) was run, and 8-bit images were opened with the ‘Load image/tracking’ button. Neurites were tracked by drawing a line between the start and end points of the neurites, using the ‘add tracking’ function of the plugin. The length of the neurites was then obtained by clicking the ‘Measure Tracings’ [47,48].

Cell Area Measurement: The areas between the pillars in the soma compartment (with the dimensions of size 899 µm × 2000 µm) were chosen as regions of interest for the graphs shown in Figure 3C and Figure S5(A(i),B(i)). Briefly, images were converted to 8-bit, then a threshold was applied using Huang settings, and ‘mean area’ was measured with the ‘Measure’ function of ImageJ and converted to percentage-based values [49]. Choosing cell area measurement over cell nucleus number is conducted to ensure that only the adherent cells are counted and that errors from autofluorescence signal are minimized (Figure S7).

Relative number of neurites: Since the number of microchannels filled with the neurites is not a defining factor for the chips’ functionality solely, it needs to be correlated with the cell density [43]. Therefore, the ratio called ‘relative number of neurites’ is defined by the relative number of microchannels filled with neurites divided by the relative cell area near the microchannel (Figure 3D and Figure S5(A(ii),B(ii))). Here, the area near the microchannel was defined as the distance between pillars to the microchannels for the whole length of the axon compartment (size of 875 µm × 5400 µm).

### 2.5. Quantification and Statistical Analysis

Statistical analysis was conducted with GraphPad Prism, version 10 (GraphPad Software, San Diego, CA, USA). A two-tailed t-test was used to compare the data in Figure 2C,F. Statistical significance is indicated by one or more asterisks: ns (non-significant), * (*p* < 0.05), ** (*p* < 0.01), *** (*p* < 0.001), or **** (*p* < 0.0001). For the other graphs in Figure 3, Figure 4 and Figure S5, first, the variance analysis was performed to check the differences between the population distribution of each microfluidic chip (*n* = 3). Since there was a significant difference, the graphs were plotted by separating each chip’s measurements for neurite length and cell area coverage. Later, the differences were analyzed with a One-way ANOVA (multiple comparisons) test.

## 3. Results

### 3.1. Manufacturing of Neuron-Foil Chips

#### 3.1.1. Characterization of the Roll-to-Roll (R2R) Imprints

*Bubble-Free Imprinting*: A big challenge in the R2R process is bubble formation [44], particularly during the imprinting process. When the filling of the structures, and air bubbles can get trapped between the imprinting resin and the microfluidic features on the nickel shim, therefore, the two different microchannel designs, *straight* and *angled*, were analyzed after the R2R imprinting step via scanning electron microscopy (SEM) (Figure 2A). The top-view images of the microchannels, as well as the cross-sectional area of a single microchannel, confirmed that the different designs were successfully fabricated using the R2R UV-NIL method since the images show a homogeneous surface and edge without defects coming from air entrapment.

*Dimension Analysis*: The UV-NIL process is performed in two steps, first, the resin (here *NILcure 31*) coated foil comes in contact with the imprinting tool-nickel shim. Second, the resin fills the structures, and afterwards, it is crosslinked via UV irradiation. During the UV-initiated crosslinking process, polymers typically show shrinking effects [50,51]. This shrinkage might cause delamination of cured resin from the foil surface and therefore needs evaluation. Geometries on both nickel shim and R2R imprint microchannels’ depth and width were measured by laser confocal scanning microscopy and compared (Figure 2B and Figure S3). The comparison of depth between the nickel shim and R2R imprint showed no significant difference, indicating no shrinkage in depth (Figure 2C). However, the comparison in channel width revealed a significant difference between nickel shim and R2R imprint for the *straight* channel from 7.03 ± 0.36 µm to 8.21 ± 0.30 µm, indicating an increase in microchannel width after polymerization, because of polymer shrinkage. This difference was not observed for the *angled* microchannel, which implies this difference could be design-driven and depends on the angled junction. All observed cross-sections have widths and depths below 10 µm, which should prevent cells from entering the microchannels [52] and lead to neurite outgrowth through the size-based geometric constraints.

#### 3.1.2. Characterization of Roll-to-Roll (R2R) Lamination and Bonding of the Neuron-Foil Chips

*Lamination*: The imprinted fluidic structures were closed by laminating a flexible PET cover foil (75 µm thick) coated with the same imprinting resin to the imprint using the custom-made R2R machine. To investigate whether the counter-pressure setting in the lamination process leads to the collapse of the channel structures, phase contrast imaging was performed after the lamination process. It was revealed that for both *straight* and *angled* microchannels, no structural collapse of the soma and the axon compartments was observed because of the incorporated pillar structures in the center of the compartment (Figure 2D,E). Further, the images confirm stable and consistent lamination across the two different microchannel designs since no delamination pockets were observed in the images.

*Bonding strength*: To investigate whether the fabrication protocol of the neuron-foil chip led to a sealed chip, the bonding strength between the R2R imprint and the foil was compared to the bonding strength of commonly used PDMS-glass microfluidic channels. The peeling test revealed that the bonding strength of the neuron-foil chip is comparable to that of plasma-bonded PDMS-glass chips because no significant difference in bonding strength was observed (Figure 2F). This demonstrates that the neuron-foil chip, hence the R2R manufacturing, serves as a viable alternative to the commonly used PDMS-glass-based microfluidic system.

### 3.2. Neuron Cell Culture in Neuron-Foil Chips

To investigate the utilization of the R2R fabricated neuron-foil chips in neurite outgrowth, the reproducibility of biological replicates, under various conditions, was tested. After 8 days of on-chip culture, cell area coverage, neurite length, and relative number of neurites (number of microchannels filled with neurites in %) were analyzed by fluorescence microscopy to identify variations between different microchannel designs and cell culture flow conditions. Before the on-chip investigation, a cytotoxicity study on the cured R2R imprinting resin revealed that the material has no cytotoxic effect based on UNE-EN-ISO 10993-5 standards [53] (Figure S4).

#### 3.2.1. Neurite Outgrowth in Static Culture

As an initial proof-of-concept, a neurite outgrowth study with the glioblastoma cells U87-MG in a static condition, without fluid flow in the compartments initiated by a rocker [9,14,17,18]. Fluorescence imaging revealed the successful neurite outgrowth into the microchannels of the *straight* (top) and *angled* (bottom) design displayed (Figure 3A) by the fact that nuclei (blue) are solely found in the soma compartment while components from the cytoskeleton, F-actin (red, white arrows) are found in the microchannels. Therefore, it can be assumed that the spatial separation of cell bodies from neurites is achieved. Based on the neurite length it was observable that in static conditions there were significant differences found between the replicates of each design (Figure 3B) with average values ranging from 182.4 ± 71.9 µm (maximum length) to 69.9 ± 24.4 µm (minimum length), indicating low reproducibility, that is independent of the design. The cell area coverage (Figure 3C) reveals that, on average, 38 to 63 percent of the area is covered by the U87-MG cells, which showed no significant difference between the replicates. However, standard deviations ranging up to ±14.9% (Figure 3C, Chip 1-*Angled*) indicate high variations in cell coverage. The relative number of neurites (Figure 3D, Table S1) demonstrates a bigger variation between replicates for the *straight* over the *angled* design.

#### 3.2.2. Neurite Outgrowth in Dynamic Culture

To investigate whether lateral flow in the axon/soma compartments or flow through the microchannels improves the neurite outgrowth and biological reproducibility, the neuron-foil chips containing U87-MG cells [49,54] were placed on a rocker during the 8-day on-chip cell culture (Figure 4A). Most notably, independent of the flow regime, lateral flow (Figure 4B(i)), or flow-through (Figure 4B(ii)), in the *straight* microchannel design, no significant changes in the neurite length per replicate were observed, whereas in the *angled* design, significant differences between replicates were observed. The average neurite length values in flow-through tend to be higher than in the lateral flow condition (Figure 4C). By comparing the average neurite length between static and dynamic culture conditions, a significant increase in the neurite length of 46%, in the flow-through condition, was observed. This indicates that the fluid flow through the microchannels slightly enhances the neurite outgrowth length. These results indicate that a *straight* microchannel design with culture media flowing through the microchannels improves both average neurite length and biological reproducibility. Further parameters, such as cell area coverage and relative number of neurites, did not show improvement in reproducibility in relation to the two flow conditions (Figure S5, Table S1). The visual observation of different cell morphologies shows that, even with additional fluid flow, the geometric constrain holds and allows the separation of the cell body (blue, nuclei) from the neurites in the microchannels (red, F-actin) indicated by the white arrows, which is comparable to the morphologies found in the static culture (Figure 3C).

## 4. Discussion

Microfluidic devices have significantly advanced axonal outgrowth studies by providing controlled microenvironments that facilitate the geometric separation of neuronal cell bodies from their neuritis, leading to improved alignment and growth rates [3,7,9,11,14,17,18,55]. Despite the high number of studies in the literature, the variety of fabrication methods is limited to a few, such as soft lithography for PDMS [7], which lacks the possibility for large-area high-throughput manufacturing. Commercially available thermoplastic microfluidic devices for neurite outgrowth studies, like Xona Microfluidics (XonaChips^®^, Durham, NC, USA) [13], are limited to the microscope slide format, which requires individual chip handling [13,43]. While some well-plate-format products exist, their primary focus lies in bigger geometries utilized in simple organ-on-a-chip applications and are not suitable for neuron outgrowth studies [33,56].

In this study, we demonstrated for the first time, to our knowledge, the high-throughput R2R manufacturing of foil-based microfluidic chips (neuron-foil chips) tailored for neurite outgrowth applications, which is the first study that uses solely the R2R UV-NIL method. Our custom-made R2R system features a large-area imprinting capability, utilizing a shim size of 630 mm × 280 mm × 250 μm, accommodating the *straight* and *angled* designs for the neuron-foil chip. Employing PET thermoplastic foils in combination with a custom-formulated UV-curable liquid resin led to the absence of air entrapment and still allowed the fine-tuning of parameters without the necessity of material changes. Notably, the PET foil substrate maintains its transparency post-crosslinking of the *NILcure 31* resin during imprinting and the subsequent lamination step, which is required for microscopic observation in cell culture. However, the final neuron-foil chips exhibit considerable autofluorescence when excited with wavelengths of 488 and 532 nm (Figure S6), which is a limitation of this system. In this study, we mitigated this issue by increasing the fluorescent dye concentrations, which improved the signal-to-noise ratio in the imaging process.

The results in Figure 2 provide evidence that the microchannels were fabricated without damages such as air entrapment in cavities and that there were minor design-related effects on the dimensions of the imprinted geometries [44]. Further, the R2R method achieved strong lamination comparable to that of the frequently used PDMS-glass chips (Figure 2F). Consequently, the R2R approach emerges as a viable alternative to the commonly used soft-lithography technique, facilitating microfluidic fabrication towards industrialization by enhancing throughput, which in turn leads to a reduction in cost and production time (Table S3).

Post-lamination, microfluidic chips were cut from the bulk-produced rolls to be combined with a bottomless 96-well plate (Figure 1D). This alignment of the well plate and chip with the thin PET foil at the bottom enables imaging with objectives that have short working distances, which are often necessary for achieving magnifications greater than 40×. The well-plate configuration permits up to eight neuron-foil chip units to be mounted on a single plate, enabling parallel experiments. The well-defined plate geometry is crucial for enabling robotic automation in modern cell cultivation, which in turn supports efficient high-throughput screening of cell assays. Compared to previous axonal studies [3,7,13,16,31,43], the proposed neuron-foil chips feature narrower microchannels, allowing for a higher density of microchannels per unit area. This increased channel density enables the collection of more data per chip, thereby enhancing the reliability of the results per unit. Further, it is shown that this narrower microchannel still allows neurite growth comparable to the literature (Figure 3A) [3,7,13,16,31,43].

Both *straight* and *angled* configurations were subjected to neuron cell culture tests under static and dynamic flow conditions. In the static condition, we found no impact on the design configuration. However, the results seemed unreliable because of significant variations in the biological replicates. Recognizing that fluidic flow positively impacts the neurite outgrowth behavior by affecting the cytoskeleton arrangement as well as altering the mechanical properties of the microenvironment for neuron cells and axonal alignment, we introduced hydrostatic pressure difference-based flow in two orientations within the neuron-foil chip [5,10,18,34,57]. The observed mean values of neurite outgrowth in both fluid flow conditions (Figure 4B,C) are consistent with prior studies performed by Liu et al. [34] and Taylor et al., respectively [7,16,43], and show that over time, the difference between the static and dynamic cultivation increases (Figure S7). Notably, in our chips, neurites did achieve comparable lengths within 8 days, which indicates an accelerated elongation rate. Additionally, Babaliari et al. demonstrated that fluidic flow direction and associated stress affect cell proliferation and found that flow direction parallel to microchannels (flow-through) is more beneficial for cell proliferation, which aligns with the increased mean values in dynamic conditions found in Figure 4B and Figure S8 [5]. It was previously reported that the design of the microchannels can affect cell morphology and their ability for neurite growth [7,13,43]. In our study, a 36% increase in average neurite length was observed in *angled* channels compared to the *straight* microchannel configuration in flow-through conditions.

A possible explanation for the increased neurite length with the flow-through condition for *angled* microchannels is the turning wall angles at the entrance of the microchannels. Recently, the number of studies focused on the axonal orientation through different wall angles increased [49,54,58,59,60]. Particularly, Girardi et al. discussed that the angle at the end of the microchannels affects the axonal behavior in terms of either sprouting or tracking the side walls [61]. To confirm their findings, additional tests such as immune staining and channel angle degrees would be required, which goes beyond the scope of this study.

In conclusion, our findings demonstrate that R2R manufacturing is a viable method for the high-throughput fabrication of microfluidic chips. The resulting foil-based chips are biocompatible platforms capable of directing neurite outgrowth, offering a valuable contribution to the study of axonal injury and regeneration. We demonstrated the validation of a manufacturing process resulting in microfluidic devices suitable for use in a standard cell culture environment. It was demonstrated that geometric constraints in the devices induce neurite outgrowth and that the reproducibility of the biological results and neurite length were dependent on fluid flow and chip design. By conducting comprehensive on-chip studies that incorporate chemical and electrical stimuli, the potential of R2R-manufactured neuron outgrowth devices can further improve their widespread application towards use in research and clinical settings.

## Data Availability

Microscopy data reported in this paper will be shared by the lead contact upon request. Any additional information required to reanalyze the data reported in this paper is available from the lead contact upon request.

## Supplementary Materials

The following supporting information can be downloaded at https://www.mdpi.com/article/10.3390/mi16060713/s1. Figure S1: Design of the neuron-foil chips. Figure S2: Computational fluid dynamics results in *straight* and *angled* microchannels. Figure S3: Characterization of microchannel *angled* and straight structures. Figure S4: Impact of R2R imprinting resin on cell viability. Figure S5: Cell area coverage and relative number of neurites in the dynamic condition. Figure S6: Autofluorescence measurement results. Figure S7: Neurite length measurements on day 4 and day 8. Figure S8: Pooled cell area coverage comparison for *straight* and *angled* microchannels for different culturing conditions. Table S1: Number of neurites for each chip, design, and flow conditions. Table S2: Reagents and devices that are used in this study. Table S3: Calculation for yield comparison of R2R UV-NIL and injection molding method.
