# Supplementary material for: Roll-to-Roll (R2R) High-Throughput Manufacturing of Foil-Based Microfluidic Chips for Neurite Outgrowth Studies
Source: Micromachines (Basel). 2025 Jun 16;16(6):713. doi: 10.3390/mi16060713 (PMC12195005; doi:10.3390/mi16060713)
Supplement: Supplementary file 1 [file micromachines-16-00713-s001.zip › micromachines-3642549-supplementary.pdf]

Supplementary Materials

Roll-to-Roll (R2R) High-Throughput Manufacturing of Foil-Based Microfluidic Chips for Neurite Outgrowth Studies

Nihan Atak[1,2], Martin Smolka[1], Anja Haase[1], Alexandra Lorenz[2], Silvia Schobesberger[2], Stephan Ruttloff[1], Christian Wolf[1], Ane Ayerdi-Izquierdo[3], Peter Ertl[2], Nerea Britz Iceta[3], Jan Hesse[1*], Martin Frauenlob[2]**

[1]Joanneum Research Forschungsgesellschaft mbH, Materials, Franz-Pichler-Strasse 31, Weiz, Austria
[2]Faculty of Technical Chemistry, TU Wien, Getreidemarkt 9, 1060 Vienna, Austria
[3]TECNALIA, Basque Research and Technology Alliance (BRTA), Mikeletegi Pasealekua 2, 20009 Donostia-San Sebastián, Spain
[*]Correspondence: jan.hesse@joanneum.at
[**]Correspondence: martin.frauenlob@tuwien.ac.at

- Figure S1: Design of the neuron-foil chips.
- Figure S2: Computational fluid dynamics results in straight and angled microchannels.
- Figure S3: Characterization of microchannel angled and straight structures.
- Figure S4: Impact of R2R imprinting resin on cell viability.
- Figure S5: Cell area coverage and relative number of neurites in dynamic condition.
- Figure S6: Autofluorescence measurement results.
- Figure S7: Neurite length measurements on day 4 and day 8.
- Figure S8: Pooled cell area coverage comparison for straight and angled microchannels for different culturing conditions.
- Table S1: Number of neurites for each chip, design, and flow conditions.
- Table S2: Reagents and devices that are used in this study.
- Table S3: Calculation for yield comparison of R2R UV-NIL and injection molding method.

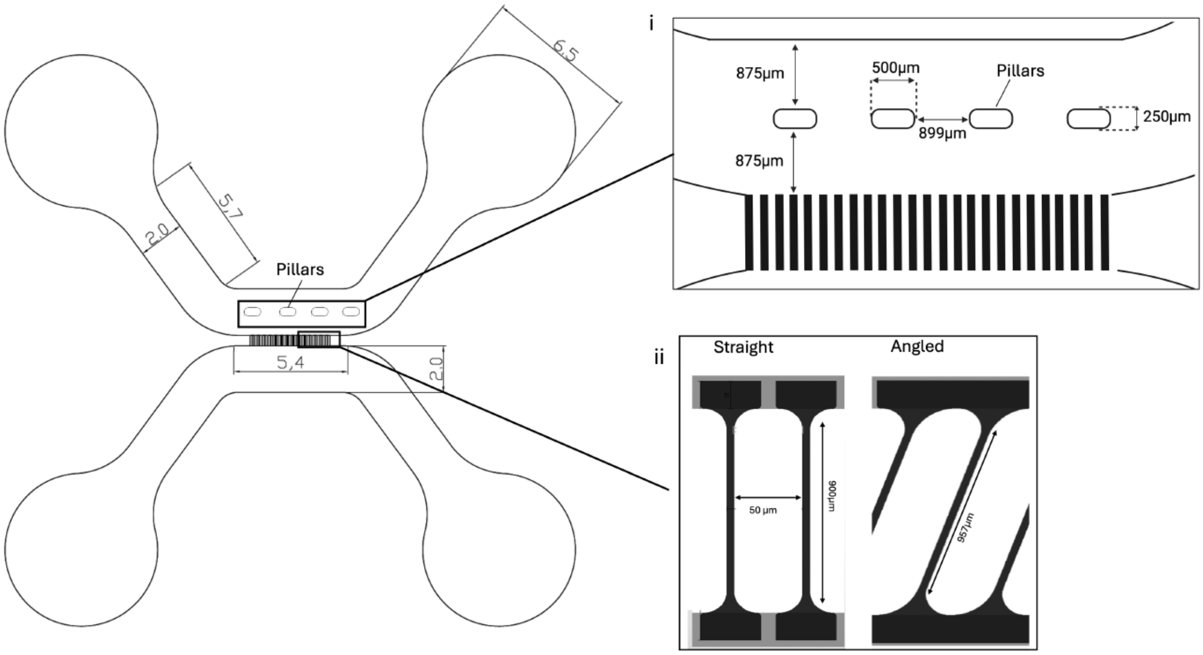

Figure S1: Design of the neuron-foil chips. Design and measures (left in cm, right in µm) of the overall chip, the pillar structure (i), and the straight and angled design of the microchannels (ii).

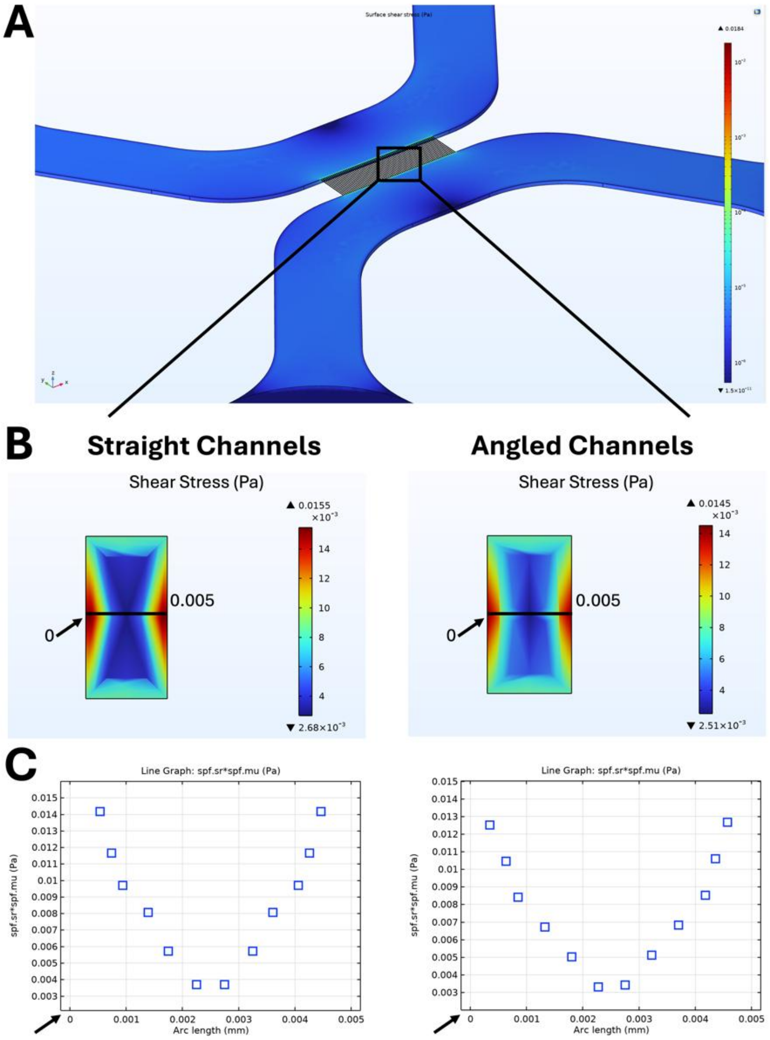

Figure S2: Computational fluid dynamics results in straight and angled microchannels. A computational fluid dynamics simulation was performed via COMSOL MULTIPHYSICS 6.1 to test if the microchannel designs, angled and straight, impact the wall shear stress. Therefore, in a 3D physics model using a stationary single-phase laminar flow, the fluid properties of water at 25°C and no slip at the wall were assumed. With an inlet to outlet pressure difference of 6,63 Pa coming from the hydrostatic height difference between the cell culture media reservoirs at 2° tilt on the rocker, the shear stress was calculated and visualized. (A) The overall single-chip shear stress heatmap focuses on the microchannel structure. The heatmap of the channel cross-section (B) and the line graphs (C) of the *straight* (left) and *angled* (right) configuration demonstrate a negligible impact of the design on shear stress because maximum values of around 0,013 - 0,014 Pa are achieved in both designs. Black arrows indicate the start location of the cut line for the line graph.

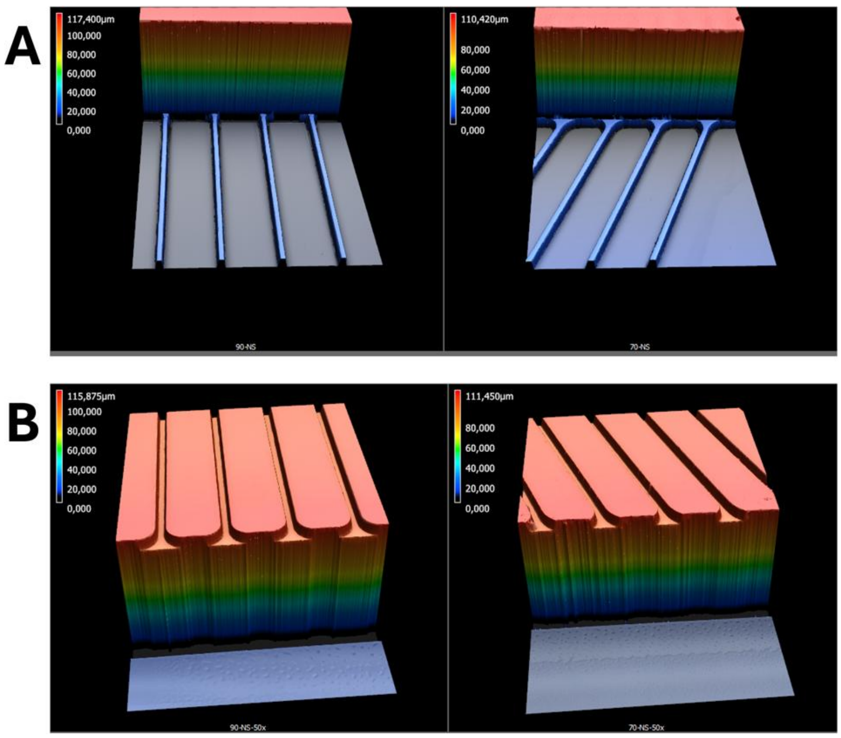

Figure S3: Characterization of microchannel angled and straight structures. The 3D laser scanning microscopy images (VK-X1050, KEYENCE, 50x) of straight (left) and angled (right) microchannel design on the nickel shim (A) and R2R imprint (B) (Sample size is 205 µm x 275 µm). Scale bars: (A) 117.40 µm (left), 110.40 µm (right). (B) 115.88 µm (left) and 111.45 µm (right).

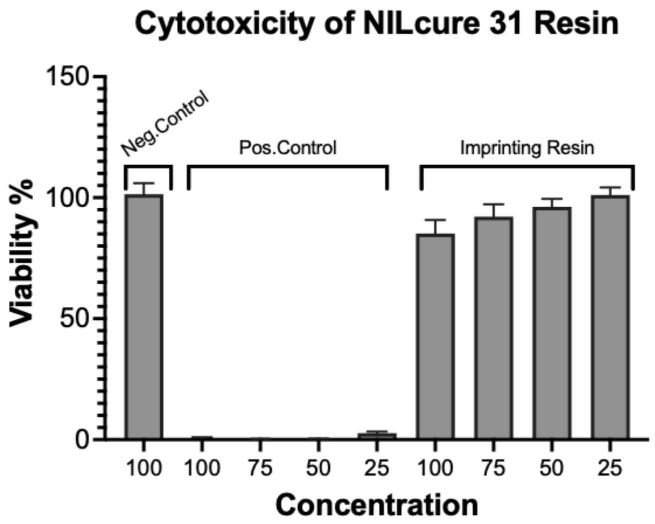

Figure S4: Impact of R2R imprinting resin on cell viability. The cured/imprinted *NILcure 31*, like other UV curable resins, is composed of monomers, crosslinkers, and photoinitiators, which potentially have cytotoxic effects on cells. Therefore, cell viability was evaluated utilizing a WST-1 cell proliferation assay. The test was performed according to the UNE-EN-ISO 10993-5 cytotoxicity standard test. Briefly, the imprinted foil from the R2R machine and the reference foils (defined by ISO) were incubated in a petri dish in cell culture media overnight. On the next day, the conditioned culture media from the sample containers were collected and diluted with unconditioned culture media in a range of 100% (undiluted) to 25% (v/v). NE-4C cells were cultured with the differently diluted conditioned cell culture media for 24h, then the WST-1 reagent was added to the cells and incubated for 3 hours. The viability evaluation performed via optical quantification in a plate reader (Cytation 1, Agilent) reveals a weak concentration-dependent effect, showing that an increase in resin concentration leads to a decrease in viability. However, *NILcure 31* was found to be *non-toxic*, since all the viability results for the UV-NIL samples are exceeding 80% (as defined by the UNE-EN-ISO 10993-5 standard).

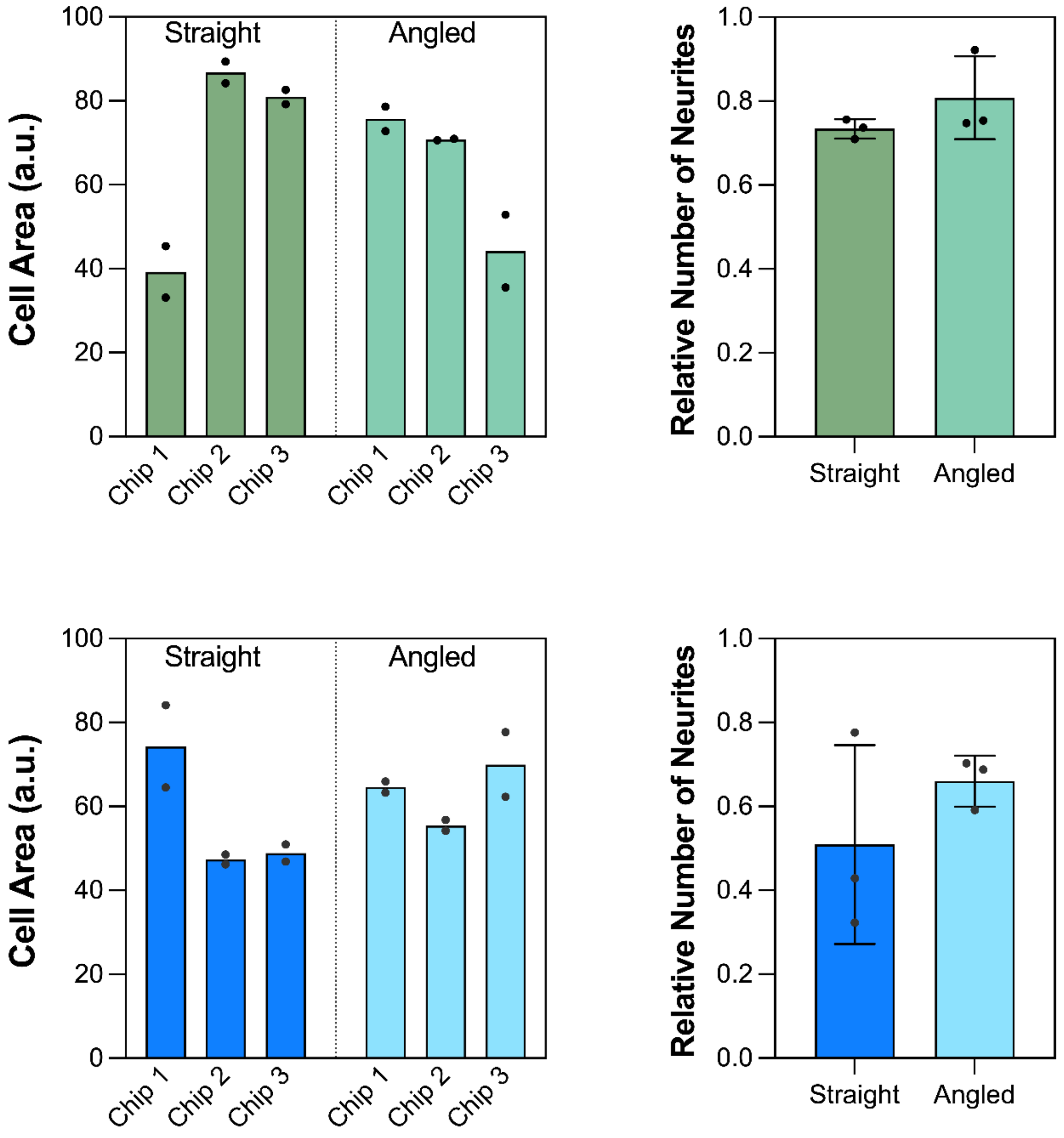

Figure S5: Cell area coverage and relative number of neurites in dynamic condition. (A) Lateral flow and (B) flow through measurement comparisons for cell area (i) and relative neurite number (ii). Data points represent the mean values ± SDs (n=3).

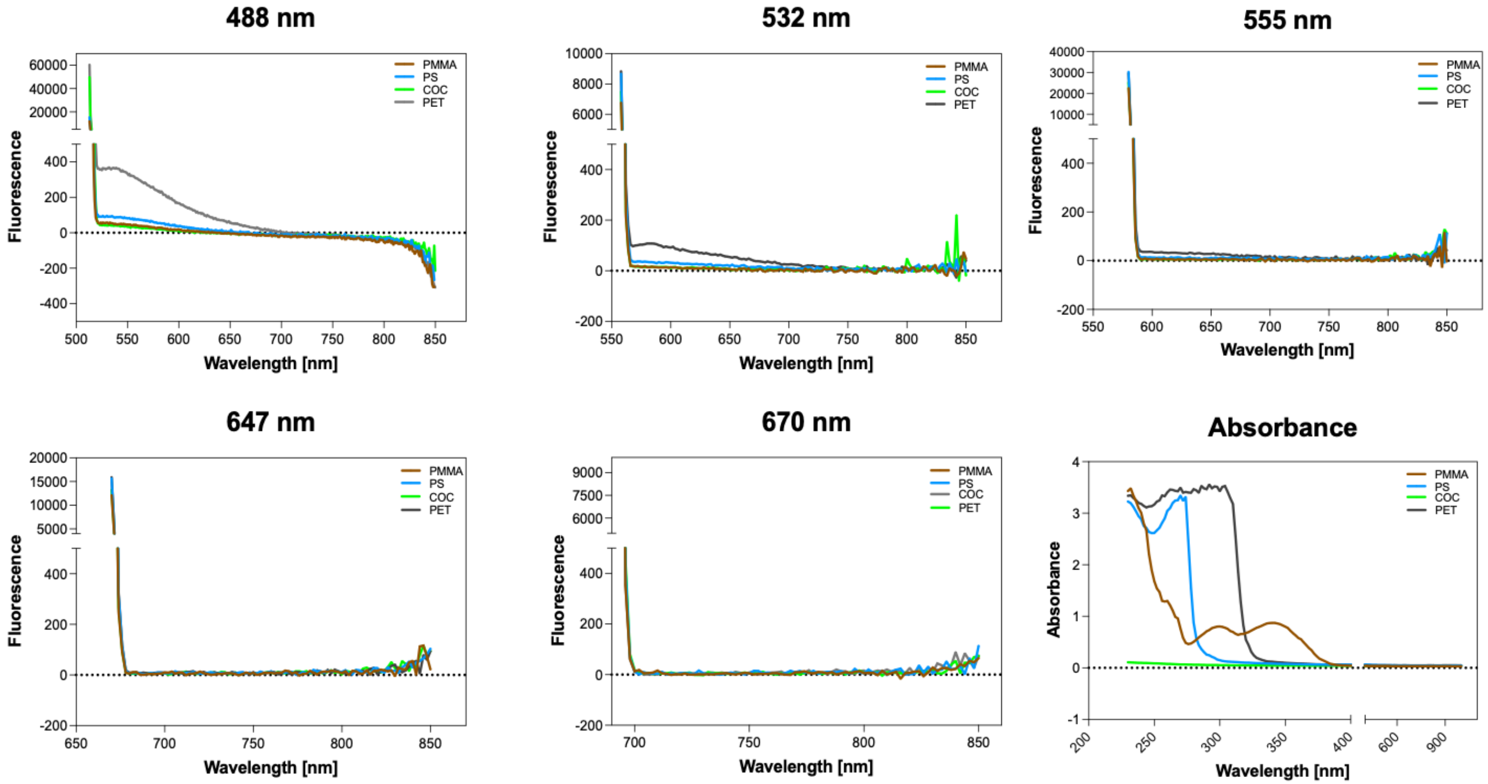

Figure S6: Autofluorescence measurement results. Autofluorescence measurements for four polymers used in microfluidic fabrication and cell culture: PS, PMMA, COC, and PET, at five different excitation wavelengths (488, 532, 555, 647, and 670 nm) and absorbance measurements for the wavelength from 200 nm to 900 nm (bottom right) using a Tecan Infinite 200 PRO reader.

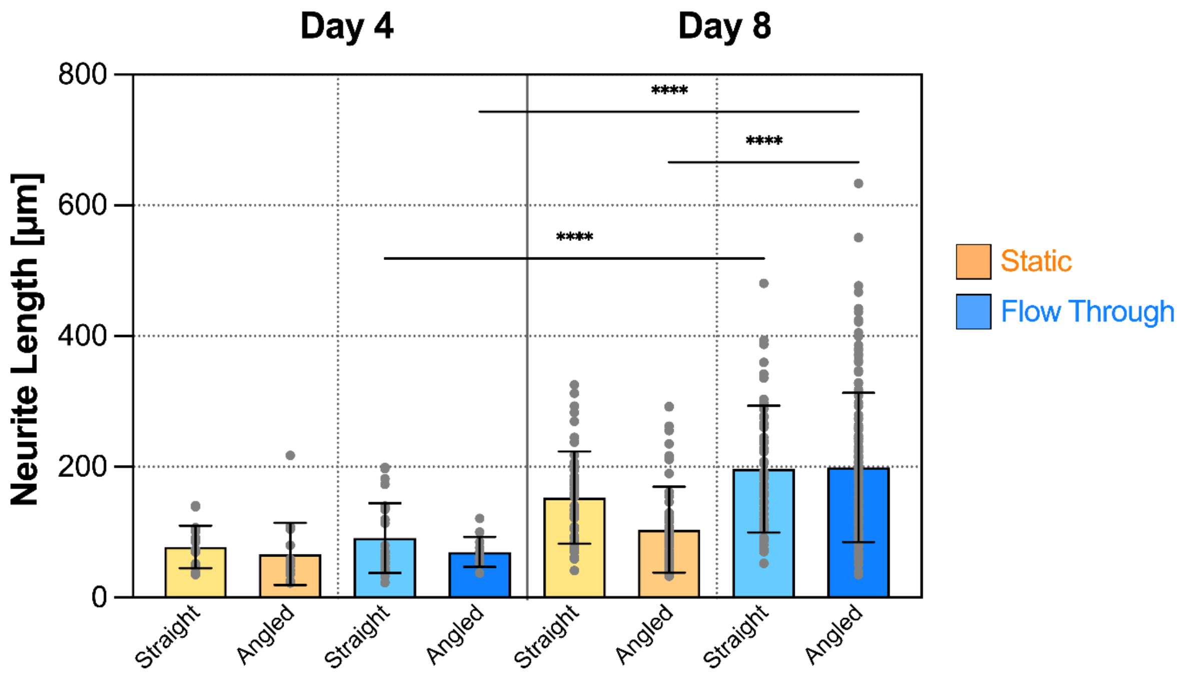

Figure S7: Neurite length measurements on day 4 and day 8. Data visualization for neurite length (grey dots represent lengths of single neurite) on day 4 and day 8. Neurite lengths were compared by microchannel design (straight and angled) for static (yellow, orange) and flow-through (light blue, dark blue) conditions on both days. It is shown that the increase in neurite length between days 4 and 8 was higher for the flow-through condition compared to the static condition. (Number of neurites: n=12-139 from 2-3 biological replicates). ****$p<0.001$.

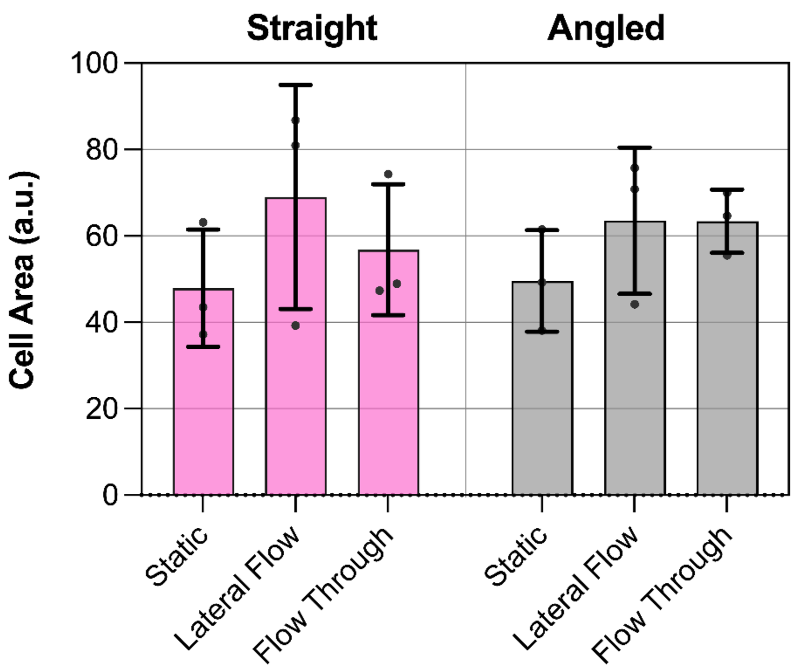

Figure S8: Pooled cell area coverage comparison for straight and angled microchannels for different culturing conditions. Cell area coverage comparison between straight (left) and angled (right) microchannels for the static and dynamic conditions (lateral flow and flow through). Data points represent the mean values ± pooled SDs (n=3 biological replicates).

Table S1: Number of neurites per chip experiment, design, and flow conditions.

	Static			Lateral Flow			Flow Through		
	Chip 1	Chip 2	Chip 3	Chip 1	Chip 2	Chip 3	Chip 1	Chip 2	Chip 3
Straight	12	30	10	33	40	60	21	23	22
Angled	22	19	22	33	49	50	53	42	50

Table S2: Reagents and devices that are used in this study.

REAGENT OR RESOURCE	SOURCE	IDENTIFIER
**Antibodies**		
Phallodin-iFluor 555	Abcam	Cat.# ab176756
Hoechst 33342	Thermo Scientific™	Cat.# 62249
**Chemicals, peptides, and recombinant proteins**		
DMEM high glucose	Sigma-Aldrich	Cat.# D6429
Collagen type 1 from rat tail	Sigma-Aldrich	Cat.# C3867
MEM NAA (100x)	Gibco	Cat.# 11140-050
Antibiotic Antimycotic Solution (100x)	Sigma-Aldrich	Cat.# A5955
PBS without $MgCl_2$	Sigma-Aldrich	Cat.# D8537
PBS with $MgCl_2$ and $CaCl_2$	Sigma-Aldrich	Cat.# D8662
FBS	Sigma-Aldrich	Cat.# F9665
EDTA-Trypsin 0,5% (v/v)	Sigma-Aldrich	Cat.# T3924
**Experimental models: Cell lines**		
U87-MG	Medical University Graz, Biobank	
NE-4C	ATCC	https://www.atcc.org/products/crl-2925
**Software and algorithms**		
ImageJ	NIH	https://imagej.net/ij/index.html
Fiji	NIH	https://imagej.net/software/fiji/
NeuronJ	Erik Meijering, PhD	https://imagescience.org/meijering/software/neuronj/
Prism version 10	GraphPad Software	https://www.graphpad.com/scientificsoftware/prism/

CellSens Imaging Software	Olympus	https://www.olympus-lifescience.com/en/software/cellsens/
Other		
Melinex PET - ST506	Pütz GmbH + Co. Folien KG	https://www.puetz-folien.com/index.php/de/
Bottomless 96-well plates	Greiner Bio-One	Cat.# 07-000-626
NILcure 31	JOANNEUM RESEARCH Forschungsgesellschaft mbH, NILcure® Materials	https://www.joanneum.at/materials/en/products/nilcure/
CO_2 laser cutter	Trotec, Austria	Speedy-Serie, Speedy 400
Double-sided adhesive tape	Microfluidic ChipShop, Germany	Cat.# 10001388
Rocker	VWR 3D Rotator Waver	Cat.# 444-0759
Scanning Electron Microscope (SEM)	JEOL, Japan	JSM-IT100
Sputter coater - SEM	Polaron SEM Coating System	Stock #: 60715-1
Tensile Tester	Instron 3342	Stock #: 73346-3
Laser Scanning Microscope	KEYENCE, Belgium	VK-X1050
Microplate reader	Tecan, Switzerland	Infinite 200 PRO

Table S3: Calculation for yield comparison of R2R UV-NIL and injection molding method.

	**R2R Fabrication**	**Injection Molding**
**Product Format**	Well plate	
**Unit Size**	4 well plates/shim	1 well plate/molding cycle
**Shim Length**	60 cm	N/A
**Machine Running Speed**	0.5 meter/minute	2 molding cycles/minute
**Yield**	3.33 well plates/minute	2 well plates/minute
**Yield Difference**	**66.5%** more well plates/minute	
